# Editorial: Multi-omics approaches in cancer research with applications in tumour prognosis, metastasis and biosensor based diagnosis of biomarkers

**DOI:** 10.3389/fonc.2023.1168975

**Published:** 2023-03-21

**Authors:** Mintu Pal, Sudhagar Selvaraju, Raju Khan

**Affiliations:** ^1^Department of Pharmacology, All India Institute of Medical Sciences (AIIMS), Bathinda, Punjab, India; ^2^Department of Biotechnology, National Institute of Pharmaceutical Education and Research, Guwahati, Assam, India; ^3^Industrial Waste Utilization, Nano and Biomaterials, CSIR-Advanced Materials and Processes Research Institute (AMPRI), Bhopal, India; ^4^Academy of Scientific and Innovative Research (AcSIR), Ghaziabad, India

**Keywords:** solid cancer, multi-omics approaches, biomarker, tumour prognosis, biosensor

Deeper insights into the molecular mechanism of cancer progression and novel early biomarkers detection by multi-omics approaches are the key to tackling tumour progression, therapeutic resistance, and recurrence after therapy.

Over the past decades, our understanding of how cancer progression can be detected has shifted sharply from the idea of an early single biomarker detection approach to multi-layered omics datasets and large-scale omics studies at the molecular level considering that tumours are heterogeneous, dynamic entities that change over time to external cues, including therapy. Indeed, molecular alteration during cancer progression is not limited to only the mutational changes of the genome but also changes in epigenomes, transcriptomes, proteomes, metabolomes, and microbiomes levels (1, 2). Although a single-gene biomarker has already been in practice, there is a huge lack of deciphering the causal relationship between molecular signatures and the phenotyping manifestation of cancer hallmarks. In contrast, the multi-signature-omics approach has the ability to uncover the underlying molecular mechanism of phenotypic manifestations of cancer hallmarks (3, 4). Therefore, it is very important to understand the complexities of cancer beyond genomics, instead, integrating multi-omics data could provide more insights into the mechanistic details of disease biology. A number of powerful bioinformatics tools including RNA-Seq data, such as the Gene Expression Omnibus (GEO) and the Sequence Read Archive (SRA) provide large scale and comprehensive data archives and analysis of coding-transcript profiling, long non-coding RNA profiling, differential expression, and regulatory networks (5). Moreover, Oncomine, GEPIA (Gene Expression Profiling Interactive Analysis) (6), provides insight into the differential expression of biomarkers in distinct clinical factors in tumours.

Another aspect of this special issue is to highlight the recent advances and future perspective concerning nanomaterials-based and nano-composites to their direct utilization in advanced sensor device applications. A wide variety of nanomaterials-based sensors have been demonstrated with potential applications in chemical and environmental sensing, bio-sensing, bio-imaging etc (7–9). Cancer biomarkers are important indicators of the detection of tumour growth. Currently available techniques for the diagnosis of cancer such as biopsy, computed tomography (CT) scan, magnetic resonance imaging (MRI), positron emission tomography (PET), ultrasound, and endoscopy etc. although, provided promising results, but these tests are very costly, requires skilled supervision and expert for data analysis and also many of them involve invasive procedures. Biosensors are devices that are designed to detect a specific biological analyte by essentially converting a biological entity (ie, protein, DNA, RNA) into an electrical signal that can be detected and analyzed. They are used not only to diagnose and monitor disease but also to provide a prognostic approach to treatment. Effective, accurate methods of cancer detection and clinical diagnosis are urgently needed. In addition, the simultaneous detection of multi-biomarkers is essential. However, because DNA is more stable than other molecules, DNA-based biosensors can play a vital role in the clinical use of biosensor-based diagnostics. Because the promised future of nanomaterials-based materials is fast approaching, scale-up studies as well as research on fabrication processes and other practical issues related to the viable production or application of these materials are of growing importance and therefore highly attractive for publication as well. Biosensor technology has the potential to provide fast and accurate detection, reliable imaging of cancer cells, monitoring of angiogenesis and cancer metastasis, and the ability to determine the effectiveness of anticancer chemotherapy agents. In addition, due to the recent great progress and growth in biosensor technology, the Internet of Medical Things (IoMT)-assisted miniaturized biomedical electronics have emerged as a potential analytical tool to manage the disease. An optimized combination of nano-enabled biosensing, POC testing, AI support, and testing interfaced with the IoMT, emerged as very useful, not only for efficient diagnostics but also to make disease management possible at a personalized level (**Figure 1**). Six papers published in this issue of *Frontiers in Oncology* focus on different perspectives by highlighting biomarkers to improve the precision of diagnostic and therapeutic methods in medicine.

**Figure 1:**
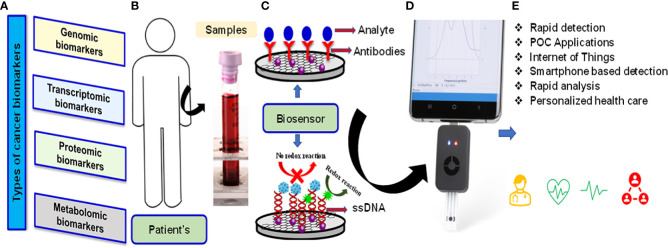
Types of cancer biomarkers **(A)** Patients samples **(B)** Schematic of biosensor-based diagnostics and procedure and mechanism of transducers **(C)** Smartphone based POC applications **(D)** Device support for AI and IoMT applications **(E)**.

Chen et al. explained Sterile alpha motif (SAM) and Src homology-3 (SH3) domain-containing 3 (SASH3) is an adaptor protein expressed mostly in lymphocytes and plays significant roles in T-cell proliferation and cell survival. Forced expression of SASH3 inhibited lung adenocarcinoma (LUAD) cell proliferation and migration. It is suggested that SASH3 could be a biomarker for the prognosis and diagnosis of human cancer. In a separate study, Chen et al. delineate the potential role of Long noncoding RNAs (lncRNAs) in the diagnosis and prognosis of Lung adenocarcinoma (LUAD). LncRNA-AC099850.3 is a novel lncRNA that is abnormally expressed in diverse cancer types including Lung adenocarcinoma (LUAD). Higher expression of AC099850.3 in LUAD is associated with an advanced tumour stage, poor prognosis, and immune infiltration. In another study, Ren et al. stated Lung cancer immune cell infiltration-associated RNA (LCIIAR) is a long noncoding RNA (lncRNA), which is overexpressed in human Lung Adenocarcinoma. LCIIAR is mainly involved in the regulation of the immune response and elevated expression of LCIIAR positively correlated with immune infiltration and immune modulator in Lung Adenocarcinoma Increased expression of LCIIAR correlated with poor clinical stage and could serve as an independent unfavorable prognostic factor in patients with Lung Adenocarcinoma. Gao et al. have revealed that high expression of 2’,5’-oligoadenylate synthetase (OAS) OAS gene family is closely related to poor prognosis of pancreatic cancer that might serve as the potential biomarker and could be the therapeutic target of pancreatic cancer. Another study by Li et al. has shown higher expression levels of ENY2 could promote cell cycle progression, cell proliferation, migration, and invasion. In addition, it is also involved in telomere maintenance, one of the fundamental hallmarks of Hepatocellular carcinoma (HCC). Li et al. systematically analyzed the relationship between DNA damage repair (DDR) alterations and non-small lung cancer (NSCLC) prognosis, and successfully validated a six-DDR gene prognostic model *via* LASSO Cox regression analysis. Based on the expression of prognostic-related DDR genes, CDC25C, NEIL3, H2AFX, NBN, XRCC5, and RAD1, the authors proposed a panel of gene signatures for hierarchical management of patients and personalized clinical treatment for NSCLC. Overall, these findings underscore the novel biomarkers by pan-cancer multi-omics approach to facilitate the treatment complexities of solid cancer. Importantly, these highlighted novel inputs could provide exciting routes for further translational research.

Even with improved understanding and documentation on cancer progression and detection at early stages, there are a number of unmet challenges in the future. One major obstacle is the integration of multi-omics data in clinical implementations, and there is a huge gap between a large volume of data generation and data processing capacity. Hence, multidisciplinary technical experts and computational advancements are required for the efficient processing of omics data, as data portals provide a repository of raw RNA-seq data, and its interpretation is crucial for the translatability of theoretical findings. The development of uniform algorithmic frameworks for processing raw omics datasets in an effective way to reveal cancer subtypes, and disease progression, and identify key drivers for genomic alterations may eventually aid the treatment strategy of individual patients in the future.

## Author contributions

All authors listed have made a substantial, direct, and intellectual contribution to the work, and approved it for publication.
